# Physical frailty characteristics have a differential impact on symptoms as measured by the CAT score: an observational study

**DOI:** 10.1186/s12955-018-0969-9

**Published:** 2018-07-16

**Authors:** Francesc Medina-Mirapeix, Roberto Bernabeu-Mora, Luz María Giménez-Giménez, Pilar Escolar-Reina, Mariano Gacto-Sánchez, Silvana Loana de Oliveira-Sousa

**Affiliations:** 1 Department of Physical Therapy, University of Murcia, Murcia, Spain; 2 Division of Pneumology, Department of Physical Therapy, Hospital Morales Meseguer, University of Murcia, Avda, Marqués de los Velez s/n, 30008 Murcia, Spain; 3 Division of Clinical Psychology, Hospital Morales Meseguer, Murcia, Spain; 4 Department of Physical Therapy, EUSES University of Girona, Girona, Spain; 5 Centre for Translational Research in Physiotherapy, Department of Pathology and Surgery, Miguel Hernández University (Elche), Alicante, Spain

## Abstract

**Background:**

The physical frailty status affects the health status of patients with chronic obstructive pulmonary disease (COPD). The objective was to determine if the individual physical frailty characteristics have a differential impact on the CAT score.

**Methods:**

This observational study included 137 patients with stable COPD. Physical frailty was measured with unintentional weight loss, low physical activity, exhaustion, slow walking speed and low grip strength and health status assessed with the COPD Assessment test (CAT). The following variables were evaluated as potential determinants of CAT: sex, age, body mass index, smoking, dyspnea, exacerbations, comorbidities, %FEV1, %FVC, anxiety and depression.

**Results:**

The prevalence of characteristics for individual frailty was as follows: low grip strength, 60.6%; low physical activity, 27.0%; exhaustion, 19.7%; slow walking speed, 9.5%; and unintentional weight loss, 7.3%. A total of 17.5% of the patients were non-frail, 73.7% were pre-frail and only 8.7% were frail. One of the five frailty characteristics, exhaustion (adjusted β coefficient 5.12 [standard error = 1.27], *p* = 0.001) was an independent determinant of CAT score in the final regression model which was adjusted by other independent determinants of CAT (dyspnea, exacerbations and anxiety).

**Conclusions:**

Due to the fact that exhaustion is a frequent and relevant psychological symptom on CAT score of patients with COPD, interventions should reduce that stress. Future research should explore how exhaustion persists or remits over time.

## Background

Chronic obstructive pulmonary disease (COPD) is characterized by persistent respiratory symptoms and airflow limitation usually caused by significant exposure to noxious particles or gases [1]. Although airflow obstruction is a strong marker of disease, the most recent recommendations of the Global Initiative for Chronic Obstructive Lung Disease (GOLD) guidelines indicate that the assessment and management of the disease should be focused on the patient’s health status, especially since poor health status is a predictor of exacerbations [2] and mortality [3]. The COPD assessment test (CAT) has been the most commonly recommended questionnaire to assess health status in patients with COPD due to its simplicity in comparison to previous and more complex tools [1, 4–7].

Several aspects have been identified in the literature as determinants for increases in the CAT score such as forced expiratory volume in 1 s (FEV1) [4], comorbidities [4, 8], and physical frailty [9]. Frailty is characterized by a decline in physiologic reserves in multiple systems and the inability to respond to stressful insults [10]. Although several conceptual frameworks have been used for describing frailty in people with COPD [9, 11–13], the Fried phenotype model is the most usual. According to this well-established and validated model, frailty encompasses five individual physical characteristics; unintentional weight loss, low physical activity, exhaustion, slow walking speed, and low grip strength [14]. The presence of one or more characteristics determines the patients’ frailty status (i.e. pre-frailty or frailty). In a recent systematic review, a percentage of 19% of patients with COPD were classified as frail and 56% were identified as pre-frail [15].

Research on frailty status and CAT scores are still scarce [9] and to our knowledge the differential impact of the individual physical frailty characteristics on the CAT score remains unexplored. If individual physical frailty characteristics can determine the patients’ health status, it could be clinically useful in the identification of patients with COPD who might benefit from specific interventions.

Therefore, the objective of this study was to determine if the individual physical frailty characteristics have a differential impact on the CAT score. Two additional secondary objectives were: 1) to explore the relevance of frailty status (i.e. pre-frail or frail) as a clinical determinant of CAT score after adjustment for other clinical determinants; 2) to identify factors associated to those individual frailty characteristics more determinants of CAT.

## Methods

### Study design and participants

A cross-sectional design was used. Patients with stable COPD were prospectively recruited from an outpatient pulmonary service at Morales Meseguer General Universitary Hospital, Murcia, Spain. The diagnosis of COPD and the stage of disease were based on the Global Initiative for Chronic Obstructive Lung Disease (GOLD) guidelines [1]. Patients with COPD, aged 40–80 years, were eligible to participate when they displayed a post-bronchodilator ratio of forced expiratory volume in 1 s/forced vital capacity (FEV1/FVC) < 0.70. Patients with an unstable cardiac condition within 4 months of the start of the study, as well as those with cognitive deterioration or unable to walk were excluded. During a 1-year period, a consecutive sample of eligible patients was identified from patient health examinations. A pulmonary physician assessed the eligibility criteria for recruitment. The institutional review board of the hospital, the “Ethical Committee of Clinical Research of the General University Hospital”, approved the study protocol (approval number: EST-35/13) and informed consent was obtained from all study participants prior to study entry. The study sample included 137 patients.

### Measures

Data were collected during face-to-face interviews and through hetero-administered questionnaires, from January to December 2015. Functional tests were performed to obtain clinical data, and other relevant clinical information was extracted from the patients’ clinical history.

### Frailty

Frailty was defined using the Fried phenotype model [14], which is well established and was validated in large epidemiological studies [16]. It consists of five features that reduce physiologic reserve and precipitate a state of vulnerability; unintentional weight loss, low physical activity, exhaustion, slow walking speed, and low grip strength. All cut-off points used were according to the original values of Fried [14]: unintentional weight loss equal or ~ greater than 4.6 kg of body weight in the last year assessed by collecting information from the medical record; low physical activity identified by the Spanish Short Version of the Minnesota Leisure Time Physical Activity Questionnaire (VREM) [17], in the lower quintile adjusted by sex; exhaustion identified by two questions from the CES-D scale [18], with a positive answer to one of the two questions; slow walking speed, assessed by 4-m gait-speed [19], lower than the 20th percentile and adjusted for sex and height; and low grip strength assessed by handgrip dynamometer [20], less than percentil 20, adjusted for sex and BMI; Patients meeting none of the Fried criteria were considered not-frail/robust, those meeting 1–2 criteria were considered pre-frail, and those with ≥3 criteria were considered frail.

### Outcome measure

For measurement of health status in patients with COPD, we used the CAT [21]. It is a very simple, short, and sensitive questionnaire used to evaluate and quantify the impact of the symptoms of COPD on patients’ health. It consists of eight items measuring different domains of respiratory health in patients with COPD (cough, phlegm, chest tightness, fatigue climbing stairs, household activities, trust/security to leave home, snoring and energy). The resulting score out of 40 indicates disease impact, with higher scores entailing worse health status.

### Demographic and health-related variables

A total of eleven variables were selected from literature research as covariates based on their potential association with either health status or CAT scores. These variables can be classified into two domains; demographic and health-related factors (clinical and psychological).

Demographic variables included sex and age (years), and were self-reported. Clinical variables included body mass index, smoking pack-years, dyspnea, exacerbations, comorbidities, percent of predicted forced expiratory volume in 1 s (FEV_1_) and forced vital capacity (FVC). They were obtained as follows: the level of dyspnea-associated activity was measured by the modified questionnaire hetero-administered and the British Medical Research Council (mMRC); spirometry was performed using standardized procedures indicated by the Spanish Society of Neumology and Thoracic Surgery; patients were measured and weighted to obtain the body mass index (kg/m^2^); and a thorough review of the patient’s clinical history, the smoking pack-years, and the number of moderate (use of corticosteroids and/or antibiotics) or serious (hospitalization) exacerbations in the previous year were considered. The psychological factors included depression and anxiety that were assessed with the Hospital Anxiety and Depression Scale (HADS) [22]. HADS is an assessment tool validated for cases of depression and anxiety in patient care consultations and in patients hospitalized with chronic diseases. The HADS consists of seven items for measuring anxiety (HADS-A) and seven items for measuring depression (HADS-D). It uses a Likert scale with four possible answers ranging from 0 to 3 points. Scores for each subscale range from 0 to 21; a score of 0–7 indicates the normal range, 8–10 is interpreted as a possible case of anxiety or depression, and 11–21 would indicate likely anxiety or depression.

### Statistical analysis

We used descriptive statistics, including Pearson χ2or Fisher’s exact test, and one way analysis of variance or Kruskal-Wallis (if non-normally distributed data), to summarize the demographic, health-related and frailty characteristics of the whole sample and the groups of patients without and with frailty status (i.e. pre-frail or frail). *P*-values under 0.05 were considered as statistically significant, except for some intermediary regression models. In these models *p* < 0.10 was used, in order to reduce the exclusion of relevant variables according to the literature research. Next, univariate and multivariable linear regression models were used to determine the association between the CAT (as dependent variable) and the demographic, health-related and individual frailty characteristics. For these analyses, several models were constructed: model 1 was unadjusted and included each one of the independent variables; model 2 included those significant demographic and health-related variables (with *p* < 0.10) from model 1; model 3 included each individual frailty characteristic after adjusting for significant (*p* < 0.10) variables from model 2; the final model adjusted for all significant (*p* < 0.10) sociodemographic, health and frailty variables simultaneously. All multivariate models were produced using the enter method, except models 2 and final, for which the backward method was used. The association between CAT and frail status (i.e. pre-frail vs non-frail) was examined in a similar way, first using an unadjusted model and second after adjusting for significant (*p* < 0.10) variables from model 2.

Finally, we also used univariate logistic regression models (by enter method) to examine relationships between patients’ characteristics and those individual frailty characteristics more determinants of CAT.

Sample size calculation was based on the rule of thumb that 15 subjects per predictor are needed for a reliable equation in multiple regression models [23]. We recruited a minimum of 120 participants assuming a maximum of 8 predictors. All analyses were performed using the Statistical Package for the Social Sciences software program (SPSS version 19.0; IBM SPSS, Chicago, IL, USA).

## Results

Of 147 patients with COPD initially enrolled, ten failed to meet our study inclusion criteria (5 with unstable cardiac condition, 3 with cognitive deterioration, and 2 unable to walk). A total of 137 patients (87.6% males) with a mean age of 66.9 (standard deviation [SD] = 8.3) years were included in our patient cohort (Table 1).

**Table 1:** Characteristics of 137 patients with COPD (Non-frail, pre-frail and frail)^a^

	All sample (*n* = 137)	Non-frail (*n* = 24)	Pre-frail (*n* = 101)	Frail (*n* = 12)	*P*-value
Demographic variables					
Age (years), mean ± SD	66.9 ± 8.3	61.1 ± 10.1	67.6 ± 7.4	72.6 ± 5.8	.001
Male	120 (87.6)	20 (83.3)	88(87.1)	12 (100)	.505
Health-related variables					
BMI (kg/m^2^)	28.9 ± 5.0	27.9 ± 4.9	29.3 ± 5.1	27.9 ± 5.1	.345
Smoking (pack-years)	58.6 ± 25.5	49.4 ± 26.3	60.7 ± 25.2	60.6 ± 25.1	.146
Dyspnea (≥ 2 mMRC)	49 (35.8)	4 (16.7)	35 (34.7)	10 (83.3)	.001
Exacerbations^b^ (≥ 2 in last year)	79 (57.7)	15(62.5)	59 (58.4)	5 (41.7)	.520
Comorbilidities (≥ 4)	50 (36.5)	1(4.2)	43(42.6)	6 (50)	.001
FEV_1_% predicted, mean ± SD	50.2 ± 16.4	49.8 ± 14.5	51.1 ± 17.1	43.0 ± 13.8	.269
FVC % predicted, mean ± SD	66.6 ± 18.7	71.4 ± 16.7	66.7 ± 19.1	56.8 ± 17.1	.084
Anxiety (HADS-A ≥ 11)	17 (12.4)	2 (8.3)	14 (14.1)	1 (8.3)	.907
Depression (HADS-D ≥ 11)	13 (9.5)	1 (4.2)	10 (10)	2 (16.7)	.477
Frailty characteristics					
Unintentional weight loss	10 (7.3)	0	6 (5.9)	4 (33.3)	
Low physical activity	37 (27.0)	0	26 (25.7)	11 (91.7)	
Exhaustion	27 (19.7)	0	21 (20.8)	6 (50.0)	
Slow walking speed	13 (9.5)	0	8(7.9)	5 (41.7)	
Low grip strength	83 (60.6)	0	71 (70.3)	12 (100)	
CAT score, mean ± SD	14.1 ± 7.3	11.4 ± 5.7	14.4 ± 7.2	18.4 ± 9.3	.021

Abbreviations: *SD* standard deviation, *BMI* body mass index, *mMRC* modified British Medical Research Council, *HADS-A* Hospital Anxiety and Depression Scale (Anxiety), *HADS-D* Hospital Anxiety and Depression Scale (Depression), *FEV1* forced expiratory volume in 1 s, *FVC* forced vital capacity, *CAT* COPD assessment test

^a^Data represent *n* (%) unless otherwise noted

^b^Moderate or severe exacerbations

A total of 57.7% of the sample reported having had two or more moderate or severe exacerbations in the past year, and 12.4 and 9.5% had anxiety and depression, respectively. According to the classification of the Fried phenotype 24 (17.5%) of the patients were considered non-frail and 101 (73.7%) were pre-frail whilst only 12 (8.7%) of the patients were frail. Regarding the prevalence of individual frailty characteristics, low grip strength was met by the largest proportion of patients in both the whole sample and the groups of pre-frail and frail patients (70.3 and 100% respectively) followed by low physical activity (25.7 and 91.7% respectively) and exhaustion (20.8 and 50%). Frail and pre-frail patients had higher CAT scores and were older than no-frail patients (Table 1). They also had higher dyspnea and comorbidities.

Table 2 shows the results of the univariate and multivariate linear regressions performed to examine the relationship between CAT scores and potential determinants. Unadjusted model (model 1) showed that almost all the selected health-related variables were associated to CAT, whereas only one of the 5 individual frailty characteristics (exhaustion) showed association. Model 2, which included significant (*p* < 0.10) demographic and health-related variables from model 1, suggests that the strongest determinants were dyspnea, exacerbations, anxiety and depression. After adjusting for these determinants (model 3), exhaustion was still associated with CAT, but their effect estimates decreased with respect to the unadjusted effects. Nevertheless, these estimates of effect were similar in the final model, when adjusted by all significant variables from models 2 and 3, and only depression was no longer a significant determinant of CAT.

**Table 2:** Association between demographic, health and frailty characteristics, and CAT^a^

Variables	Models 1^c^	Model 2^d^	Models 3^e^	Final Model
Demographic variables				
Age (years)	0.03 (0.07) 0.715	N.a.		N.a.
Male	0.25 (1.90) 0.894	N.a.		N.a.
Health-related variables				
BMI (kg/m^2^)	0.23 (0.12) 0.067			N.a.
Smoking (pack-years)	0.03 (0.02) 0.140	N.a.		N.a.
Dyspnea (≥ 2 mMRC)	8.89 (1.06) 0.001	7.28 (1.08) 0.001		6.20 (1.08) 0.001
Exacerbations^b^ (≥ 2)	4.13 (1.20) 0.001	2.13 (1.02) 0.038		2.17 (0.99) 0.031
Comorbilidities (≥ 4)	2.57 (1.28) 0.048			N.a.
FEV_1_% predicted	−0.12 (0.04) 0.001			N.a.
FVC % predicted	−0.09 (0.03) 0.005			N.a.
Anxiety (HADS-A ≥ 11)	9.05 (1.74) 0.001	3.36 (1.67) 0.046		3.85 (1.51) 0.012
Depression (HADS-D ≥ 11)	8.87 (2.01) 0.001	5.45 (1.82) 0.003		
Frailty characteristics				
Unintentional weight loss	0.22 (2.41) 0.684	N.a.	0.12 (1.89) 0.948	N.a.
Low physical activity	0.11 (1.41) 0.551	N.a.	0.39 (1.08) 0.715	N.a.
Exhaustion	8.98 (1.37) 0.001	N.a.	4.26 (1.35) 0.002	5.12 (1.27) 0.001
Slow walking speed	3.61 (2.11) 0.486	N.a.	0.72 (1.65) 0.665	N.a.
Low grip strength	0.95 (1.28) 0.457	0.20 (0.95) 0.835	0.20 (0.98) 0.835	N.a.

Abbreviations: *BMI* body mass index, *mMRC* modified British Medical Research Council, *HADS-A* Hospital Anxiety and Depression Scale (Anxiety), *HADS-D* Hospital Anxiety and Depression Scale (Depression), *FEV1* forced expiratory volume in 1 s, *FVC* forced vital capacity, *CAT* COPD assessment test

^a^Data represent B coefficients (standard error) and p-value unless otherwise noted

^b^Moderate or severe exacerbations in last year

^c^Models 1 are unadjusted models of each demographic, health and frailty characteristics

^d^Model 2 includes significant (*p* < 0.10) sociodemographics and health variables from model 1

^e^Models 3 includes significant (*p* < 0.10) variables from model 2 and each individual frailty characteristic included separately

Final Model is fully adjusted and includes significant variables from models 2 and 3

N.a. Not included in the model

The association between CAT and global frail status (frail and pre-frail versus non-frail patients) was only significant for the frail group in the unadjusted model [β coefficient = 7.04, Error typical = 2.53, *p*-value = 0.006] (data not reported on Table), but it was not on multivariate analysis [β coefficient = 2.37, Error typical = 2.14; p-value = 0.272] when adjusting by the strongest clinical determinants of CAT from model 2 of Table 2 (i.e. dyspnea, exacerbations, anxiety and depression).

Table 3 shows the results of univariate and multivariate analyses to identify factors associated with exhaustion. The unadjusted analyses provide a relative measure of the likelihood of having exhaustion regardless of additional factors. Based on these unadjusted results, three variables (dyspnea, anxiety and depression) were included in the multivariate model. According to this adjusted model, the odds of being exhausted increases when patients have dyspnea (OR = 5.62; 95% CI:1.98–15.93) and depression (OR = 22.63; 95% CI:5.0–102.32).

**Table 3:** Association between demographic and health-related variables, and Exhaustion

Variables	OR univariate [95% CI]	OR multivariate [95% CI]
Demographic variables		
Age (years)	1.01 [0.96–1.06]	
Male	0.85 [0.22–3.22]	
Health-related variables		
BMI (kg/m2)	1.05 [0.96–1.14]	
Smoking (pack-years)	1.00 [0.99–1.02]	
Dyspnea (≥ 2 mMRC)	5.09 [2.06–12.55]**	5.62 [1.98–15.93]**
Exacerbations^a^ (≥2)	1.60 [0.66–3.88]	
Comorbilidities (≥ 4)	1.02 [0.43–2.46]	
FEV1% predicted	0.99 [0.97–1.02]	
FVC % predicted	0.98 [0.96–1.01]	
Anxiety (HADS-A ≥ 11)	8.48 [2.84–25.34]**	
Depression (HADS-D ≥ 11)	20.78 [5.18–83.28]**	22.63 [5.0–102.32]**

Abbreviations: *BMI* body mass index, *mMRC* modified British Medical Research Council, *HADS-A* Hospital Anxiety and Depression Scale (Anxiety), *HADS-D* Hospital Anxiety and Depression Scale (Depression), *FEV1* forced expiratory volume in 1 s, *FVC* forced vital capacity, *CAT* COPD assessment test

^a^Moderate or severe exacerbations; **p < 0.01

## Discussion

The main objective of our study was to analyze whether there is a differential impact of the individual characteristics of frailty on the CAT score. Of the five individual frailty characteristics examined, exhaustion was the only determinant of an increase in the CAT score. We also found that frail status was not associated with a higher CAT scores when this relationship was adjusted for potential confounding factors (dyspnea, exacerbations and anxiety).

In the final multivariate model, dyspnea and exhaustion had the largest effect on the CAT score. Considerable evidence demonstrates a relationship between dyspnea and health status [24]. Exhaustion may affect CAT in a similar way as dyspnea. In our opinion, the perception of exhaustion may serve as a self-protective function, leading to self-limitations during activities and to subsequent physical deconditioning [25]. In turn, it may lead to further increases in exhaustion, resulting in greater inactivity and poorer health status.

In our study, CAT was not associated with physical activity and gait speed. Previous studies have also noticed a poor relationship between CAT and physical activity [24, 26]. A possible explanation is that there is an interaction between the volume of the physical activity and the experience of the symptoms [24]. It is therefore plausible that patients can decrease their level of physical activity to avoid symptoms [24, 27]. Because some items of the CAT score are related to respiratory symptoms, patients’ perception of health status may therefore be favorable in low levels of activity. On the other hand, while there is consistent evidence that gait speed (measured in meters/second) correlates with health status [19], these relevant relationships might have been obscured by using a dichotomous method to define this criterion in the frailty model.

Our results show that frail patients have lower CAT in an unadjusted model, but not in an adjusted model. The relevance of frailty status on the CAT score has been suggested by Maddocks et al. [9]. However, Maddocks’ study used only an unadjusted model to examine the relationship between frailty and CAT score, which differed from our study. In fact, our bivariate analyses found that pre-frail and non-frail patients had different levels of pulmonary function and psychological disorders. Therefore, these dysfunctions could play a confounding role in the relationship between frailty and health status.

The prevalence of frail patients was similar to a previous study using Fried criteria that included community elderly patients with COPD [13], but was quite lower than a study including patients participating in pulmonary rehabilitation programs [9]. Usually, these programs are designed to optimize physical and social performance and autonomy in persons with COPD [28]. Since the reference hospital of our study does not have a respiratory rehabilitation unit, nobody undergoing pulmonary rehabilitation. Thus, it is possible that the differences between the prevalence reported in Maddocks’ study and our study were due to the physical or social characteristics of the two patient populations. On the other hand, a previous study also found a higher prevalence of frailty [11, 29], but this result is not directly comparable because they used another framework or model of frailty. Moreover, Park’s study [11] should be considered with caution because patients were included based on COPD symptoms and not on the results of a post-bronchodilator spirometry, as recommended by the GOLD guidelines.

In our study the “low grip strength” was the most prevalent frailty characteristic and we observed a higher prevalence than that reported in previous studies [9, 13]. The prevalence of exhaustion shown in our study was also similar to a study by Lahousse [13], that included elderly patients. However, our prevalence of exhaustion was lower than that seen in Maddocks’ study [9], in which participants of a similar age were included, despite the fact that they had more severe FEV1 compared to our population. Differences may be explained by physical or social characteristics associated with participation in rehabilitation programs, as previously stated.

Our study highlighted that a combination of symptoms and psychological factors (i.e. dyspnea and depression) are possible underpinnings of exhaustion in patients with COPD. Psychological factors have been associated with exhaustion in previous studies assessing older people albeit with other clinical conditions [30]. On the other hand, while dyspnea has been widely associated with fatigue [31, 32], to our knowledge this is the first study to identify dyspnea as a factor associated specifically with exhaustion in patients with COPD. However, among patients with COPD, a cross-sectional relationship between depression and exhaustion was also previously identified. This is understandable based on the fact that exhaustion is the feeling of extreme fatigue [30].

### Significance of the findings

The results of our study provide important aspects for clinical practice. On the one hand, because the presence of exhaustion in patients with COPD has a negative impact on the perception of their health status, it is necessary to explore it beyond other usual psychological aspects such as anxiety and depression. On the other hand, because psychological characteristics interact with physical symptoms and play an important role in exhaustion and how COPD patients experience and manage their disease [33], specific interventions must be conducted to encourage self-management and to provide disease education and health behaviors in order to reduce stress during situations that can result in psychological problems. These interventions can be performed as part of usual care provided by specialists or as part of pulmonary rehabilitation programs. The latter have also showed relevance on CAT score and other clinical outcomes [34]. Moreover they have the advantage that the approach is multidisciplinary (physician, physiotherapist, psychologist, nutritionist, etc.) and provides the proper context to simultaneously improve the physical and psychological condition of patients. Additionally, patients may benefit from contact with other patients with the same clinical condition [35].

It is important to recognize the limited scope of the present study and to consider future research directions. For example, further research could explore consequences over time of exhaustion, how this characteristic persists or remits over time, and eventual longitudinal associations between exhaustion and CAT scores.

### Limitations

To our knowledge, no previous studies have analyzed the relevance of individual frailty characteristics in patients with COPD. The inclusion of a wide variety of determinants of CAT in our models could be considered as one of the strengths of the study. Nevertheless, our study had several limitations. First, patients were excluded if they had unstable cardiac condition, cognitive deterioration or were unable to walk. This fact might have affected our findings, considering that some of the aforementionend patients could have been more frail than those included. Few patients were nonetheless excluded and, consequently, the possible bias should be low. Second, we include a wide and acceptable variability of determinants of CAT, but it may be speculated that some other factors not included in our models could even further improve its power (e.g. medication, participation in rehabilitation programs, etc) [9, 28, 36]. However, information on medication was not available and our hospital doesn’t have a respiratory rehabilitation unit. Finally, because of the small number of women in the cohort, caution should be taken when generalizing the results to women.

## Conclusions

Of the five individual frailty characteristics included in the Fried phenotype model, only exhaustion is a determinant for the health status of patients with COPD as measured by the CAT score. Exhaustion is a frequent psychological symptom in patients with COPD and therefore the clinical management of these patients should include specific interventions to help them cope with the disease and reduce prolonged stress.

## Abbreviations

BMI: body mass index
CAT: Chronic Obstructive Pulmonary Disease Assessment Test
CES-D: Center for Epidemiologic Studies Depression
CI: Confidence interval
COPD: Chronic Obstructive Pulmonary Disease
FEV_1_: Forced expiratory volume in 1 s
FVC: Forced vital capacity
GOLD: Global Initiative for Chronic Obstructive Lung Disease
HADS: Hospital Anxiety and Depression Scale
HADS-A: Hospital Anxiety and Depression Scale (Anxiety)
HADS-D: Hospital Anxiety and Depression Scale (Depression)
mMRC: modified British Medical Research Council
OR: Odds ratio
SD: Standard deviation
VREM: Spanish Short Version of the Minnesota Leisure Time Physical Activity Questionnaire
